# Eco-friendly spectrophotometric determination of moxifloxacin in pharmaceutical dosage form via ion-pair complexation with erythrosine B

**DOI:** 10.1186/s13065-026-01879-x

**Published:** 2026-07-15

**Authors:** Tamer Z. Attia, Asmaa Mohamed Abbas, Deena A. M. Nour El-Deen, Mahmoud A. Omar

**Affiliations:** 1 Pharmaceutical Analytical Chemistry Department, Faculty of Pharmacy, Minia University, Minia, Egypt; 2 Department of Pharmacognosy and Pharmaceutical Chemistry, College of Pharmacy, Taibah University, Medinah, Saudi Arabia

**Keywords:** Moxifloxacin, Erythrosine B, Ion-pair complexation, Spectrophotometry, Green analytical chemistry, Pharmaceutical analysis

## Abstract

A rapid, sensitive, and eco-friendly spectrophotometric method was developed for the quantitative determination of moxifloxacin (MOX) based on ion-pair formation with erythrosine B (EB). The resulting pink-colored ion pair complex exhibited a maximum absorbance at 554 nm. Systematic optimization of reaction parameters, including buffer pH, dye concentration, and diluent selection, ensured maximum sensitivity and reproducibility. The method demonstrated excellent linearity within the concentration range of 0.2–1.2 µg mL^− 1^ (*r* = 0.9990), with low detection and quantification limits (LOD = 0.05 µg mL^− 1^, LOQ = 0.15 µg mL^− 1^). The calculated molar absorptivity (3.41 × 10⁵ L·mol⁻¹·cm⁻¹) and Sandell’s sensitivity (1.18 × 10⁻³ µg·cm⁻² per 0.001 absorbance unit) confirmed the high analytical sensitivity of the proposed method. Validation in accordance with ICH guidelines demonstrated excellent accuracy (mean recoveries of 99.43–99.77%), precision (RSD < 1.7%), and robustness against minor variations in experimental conditions. Application of the proposed method to commercial eye-drop formulation produced recoveries ranging from 99.1% to 100.46%. Statistical comparison with reference method revealed no significant differences (*t-* value = 1.008, *F-* value = 1.124). These findings establish the proposed assay as equivalent in accuracy and precision to established techniques, while offering practical simplicity, cost-effectiveness, and environmental compatibility. By eliminating organic solvents and minimizing reagent consumption, the method aligns with green chemistry principles and provides a practical, sustainable tool for routine pharmaceutical quality control.

## Introduction

**Fig. 1:**
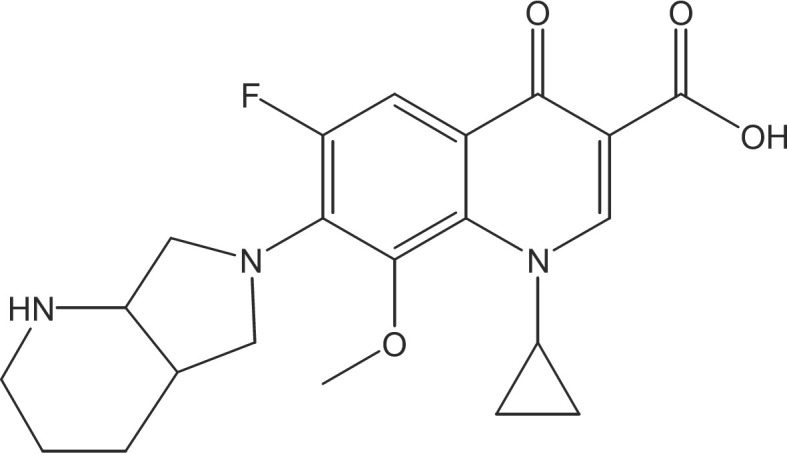
Chemical structure of moxifloxacin

Moxifloxacin (MOX, Fig. 1) is a fourth-generation fluoroquinolone antibiotic with broad-spectrum activity against both Gram-negative and Gram-positive bacteria, making it an important therapeutic agent for the treatment of a wide range of bacterial infections [1–3]. Given its widespread clinical use; reliable, sensitive, and efficient analytical methods for MOX determination are essential to ensure drug quality, patient safety, and therapeutic efficacy.

Several analytical techniques have been reported for the quantitative determination of MOX, including high-performance liquid chromatography (HPLC) [4–9], capillary electrophoresis [10, 11], spectrofluorimetric methods [12–21], and spectrophotometric methods [22–26]. Although these methods provide accurate results, many are associated with certain limitation such as high cost, complex instrumentation, time-consuming procedures, or reliance on organic solvents that raise environmental concerns. HPLC and capillary electrophoretic techniques, though sensitive, are not always practical for routine quality control in laboratories with limited analytical resources. Spectrofluorimetric methods, on the other hand, often require specialized reagents or micellar systems, which may complicate their application in pharmaceutical analysis. On the other hand, recent eco-friendly spectrophotometric approaches have demonstrated strong sustainability metrics, reinforcing the importance of solvent-free and cost-effective assays in pharmaceutical analysis [27, 28].

To overcomes these limitations, the present study reports a simple, eco-friendly, and sensitive spectrophotometric method for MOX determination based on ion-pair complex formation with erythrosine B (EB), a xanthene dye commonly used as a food colorant. The method eliminates the need for extraction steps, minimizes solvent use, and provides rapid colorimetric detection at 554 nm. Following systematic optimization of the reaction conditions (including buffer pH, dye concentration, and solvent choice), the proposed assay achieves excellent sensitivity and reproducibility. Importantly, it offers a simple and cost-effective alternative to sophisticated chromatographic techniques, making it particularly well suited for routine quality control of MOX in bulk drug substances and pharmaceutical dosage form.

## Experimental

### Apparatus

Absorbance measurements were performed using a Shimadzu UV-1900i spectrophotometer (Shimadzu Corporation, Kyoto, Japan). The device was equipped with UVProbe software (Version 3.7) and two quartz sample cells (1 cm), one for the blank solution and one for the tested sample. pH measurements were carried out using a Milwaukee SM 101 pH meter (Milwaukee Instruments, Portugal).

### Materials and chemicals

MOX pure sample (99.8%) was kindly supplied by EVA pharmaceuticals (Cairo, Egypt). Moxiflox^®^ ophthalmic solution (5 mg/ml, batch NO. KAT50Q5C85D40H, EVA pharmaceuticals) was purchased from local pharmacy in Egypt. Acetic acid was purchased from El-Nasr chemical Co., Cairo, Egypt. Erythrocin B (EB, analytical grade) was purchased from Loba Chemie, Mumbai, India and prepared as a 0.15% w/v solution in distilled water. Acetate buffer (0.2 M, pH 3.4) was prepared using glacial acetic acid (analytical grade, El-Nasr Chemical Co., Cairo, Egypt) and sodium acetate (analytical grade, El-Nasr Chemical Co., Cairo, Egypt).

### Standard solutions

In a 100 mL volumetric flask, a stock solution of MOX (100 µg mL⁻¹) was prepared by accurately weighting and dissolving 10 mg of MOX in distilled water. The working solutions were prepared by further dilution with distilled water to obtain the final concentrations ranging from 0.2 to 1.2 µg mL^− 1^. The stock solution was stable for at least two weeks when kept at 4 °C.

### General analytical procedure

An aliquot of 0.5 mL of the prepared MOX working solution was transferred into a 10-mL volumetric flask. Subsequently, 0.5 mL of 0.15% (w/v) EB solution and 2 mL of 0.2 M acetate buffer (pH 3.4) were added. The mixture was then diluted to the mark with distilled water. The absorbance of the resulting reaction mixture was measured at 554 nm against a reagent blank prepared under identical conditions at room temperature (25 ± 2 °C).

### Analysis of pharmaceutical eye-drop formulation

In a 25-mL volumetric flask, 5 mL of the eye drop solution was filtered and diluted to the mark with distilled water. The resulting solution was further diluted with distilled water to obtain a working solution of 100 µg mL^− 1^.

## Results and discussion

The development of a simple, sensitive, and eco-friendly spectrophotometric assay for MOX determination is of considerable importance in pharmaceutical analysis. Conventional chromatographic and fluorometric techniques, although sensitive, often require sophisticated and expensive instrumentation, specialized reagents, or large volumes of organic solvents, which limit their accessibility in routine quality control laboratories. In contrast, the proposed ion-pair complexation method with EB offers several analytical and environmental advantages:

- Accessibility and Cost-Effectiveness: The method relies on widely available reagents and standard UV–Vis spectrophotometer, making it suitable for resource-limited laboratories.
- Eco-friendliness: By eliminating organic solvents and extraction steps, the assay aligns with green chemistry principles, reducing environmental impact and laboratory hazards. Several recent studies have applied greenness assessment tools to evaluate spectrophotometric and related methods. For instance, Fe-doped carbon dots have been utilized in nanozyme-based colorimetric and fluorometric sensing of levodopa [29], while green microextraction combined with paper-based techniques and smartphone sensing has enabled sustainable determination of nicotinamide [30] in pharmaceuticals and biological samples. These reports underscore the importance of structured greenness evaluation metrics (e.g., GAPI, Eco-scale, AGREE) in method development. Within this framework, our solvent-free spectrophotometric assay—relying solely on distilled water and minimal reagent consumption—fits well within the framework of sustainable pharmaceutical analysis, providing validated robustness together with environmental compatibility.
- Rapidity and Simplicity: The direct colorimetric detection at 554 nm allows rapid analysis without lengthy sample preparation procedures.
- Reliability: Validation according to ICH guidelines confirms excellent sensitivity, accuracy, precision, and robustness, ensuring reliable analytical performance for pharmaceutical quality control.
- Practical Application: The method has been successfully applied to commercial eye drop formulation, as demonstrated by high recovery values with no interference from common pharmaceutical excipients.

By integrating sustainability with strong analytical performance, the proposed spectrophotometric approach offers a practical alternative to more complex techniques. It supports routine pharmaceutical quality control and encourages broader adoption of green methodologies in pharmaceutical laboratories.

As summarized in Table 1, previously reported spectrophotometric methods for moxifloxacin determination [22–26] often relied on hazardous acidic or alkaline media, complex kinetic monitoring, or multi-step derivatization procedures. These approaches not only increased analysis time and cost but also generated chemical waste and posed occupational risks. In contrast, the proposed MOX–EB ion-pair complexation method eliminates organic solvents, minimizes reagent consumption, and achieves superior sensitivity (LOD = 0.050 µg mL⁻¹) across a practical linear range. The absence of observed limitations, combined with validated robustness and strong greenness metrics (EcoScale, GAPI, AGREE), underscores its suitability as a sustainable and routine quality control tool for pharmaceutical formulations.

**Table 1:** Comparison of reported spectrophotometric methods for moxifloxacin determination versus the proposed method

Reference	Technique/principle	Linear range (µg mL^− 1^)	LOD (µg mL^− 1^)	Solvent used	Greenness assessment	Analysis time/cost	Applicability
Elbashir et al. (2013) [22]	Spectrophotometric derivatization with NQS	2.5–20	0.75	Buffer solution pH 11	Not assessed	20 min/ moderate	Commercial Tablets
	oxidation with cerium (IV)	0.5–30	0.16	1 M sulphuric acid		10 min/moderate	
Sahu et al. (2011) [23]	UV spectrophotometry	2–8	0.011	0.01 N HCI.	Not assessed	Immediate/Low	Commercial Tablets
Ashour and Bayram (2015) [25]	Kinetic spectrophotometry oxidative coupling with MBTH in the presence of Ce(IV)	1.89–40.0	0.644 and 0.043	Acidic media of sulphuric acid	Not assessed	~ 20 min/ moderate	Commercial Tablets
Abdellaziz and Hosny (2011) [26]	kinetic spectrophotometric method based on oxidation by Fe^3+^ ion in presence of 1,10 o-phenanthroline	0.8–6	0.076	1 M HCl, Water	Not assessed	35 min/high	Commercial Tablets
	reduce Fe (III) to Fe (II), complex with 2,2’ bipyridyl	0.8–4	0.21	1 M HCL, water	Not assessed	30 min/high	
	Ion- pair associated with bismuth (III) tetraiodide	16–96	1.41	HNO_3_, water	Not assessed	Complex procedures/high	
Proposed method (2026)	Ion-pair complexation with EB	0.2–1.2	0.05	Distilled water	Aligns with green chemistry principles	Immediate/Low	Eye drops

NQS: 1,2-naphtho-quinone-4-sulphonate; MBTH: 3-methyl-2-benzothiazolinone hydrazone hydrochloride monohydrate

### Optimization of analytical parameters

Optimization of the experimental variables for ion-pair complexation was essential to maximize sensitivity and reproducibility of the proposed assay. Each experimental factor was systematically examined while keeping all other variables constant, ensuring that the chromogenic complex between MOX and EB was formed under optimum experimental conditions.

#### Influence of buffer pH and volume

**Fig. 2:**
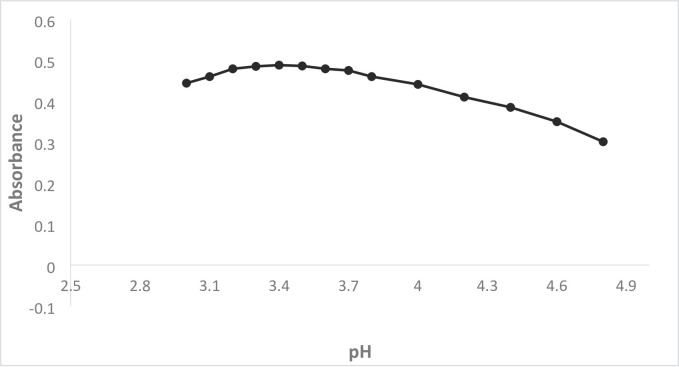
Effect of the pH on the formation of the yielded ion-pair complex of MOX (0.5 µg mL^− 1^) with EB

The acidity of the medium significantly governs the extent of ion-pair formation. Screening acetate buffer across the pH interval 3.0–5.0 (Fig. 2) revealed that pH 3.4 produced the highest absorbance response, confirming the importance of protonation in stabilizing the complex.

**Fig. 3:**
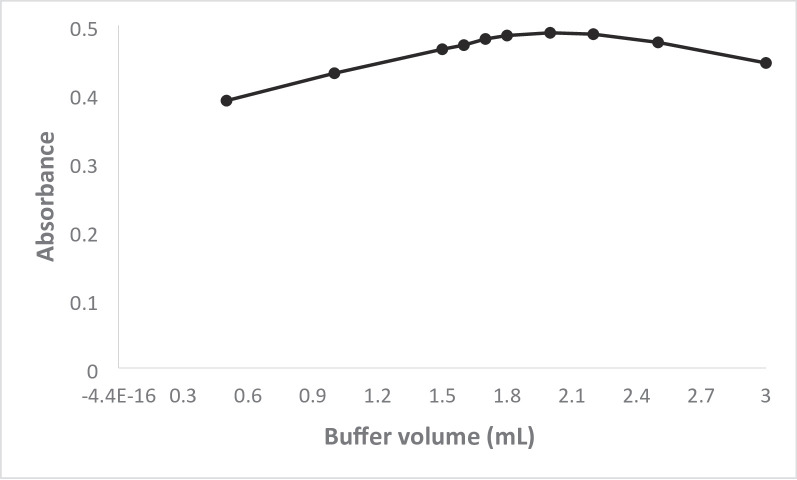
Effect of buffer volume on the formation of the complex of MOX (0.5 µg mL^− 1^) with EB

Buffer volume effect was also examined; volumes below 2.0 mL yielded weaker signals, whereas excessive buffer diluted the system. A volume of 2.0 mL was identified as the most suitable, balancing stability with reproducibility (Fig. 3).

#### Effect of dye volume

**Fig. 4:**
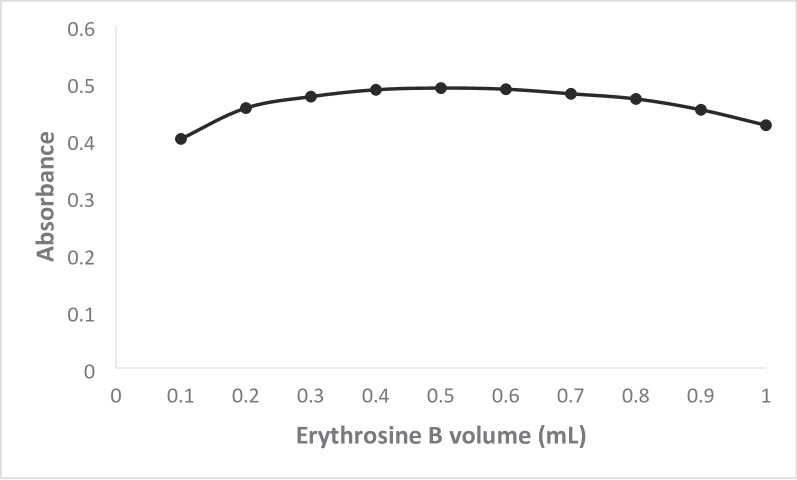
Effect of EB volume on the formation of the complex of MOX (0.5 µg mL^− 1^) with EB

The volume of EB directly affects the degree of complexation. Incremental additions of dye solution enhanced absorbance until a plateau was reached (Fig. 4). Beyond 0.6 mL, turbidity and instability appeared, indicating excess dye. Thus, 0.5 mL was selected as the optimal dye volume, providing maximum absorbance while maintaining solution clarity.

#### Temperature and reaction time

**Fig. 5:**
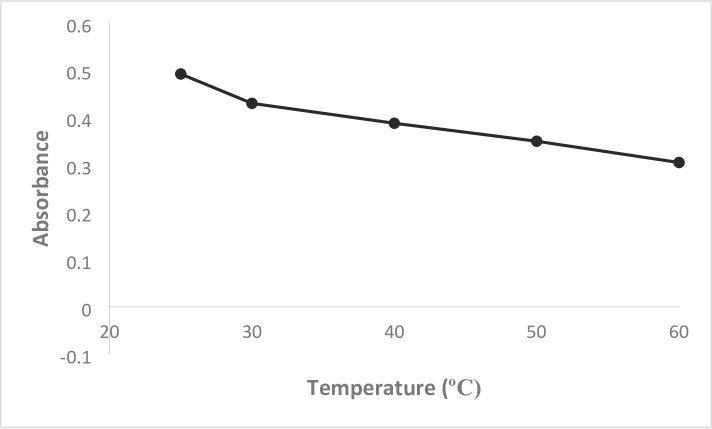
Temperature influence on the formation of the complexes of MOX (0.5 µg mL^− 1^) with EB

Monitoring the kinetics of color development demonstrated that the complex formed rapidly and remained stable for at least 20 min at ambient temperature. Elevated temperatures, however, induced precipitation and reduced absorbance, underscoring the necessity of conducting the assay under controlled room conditions (Fig. 5).

#### Effect of diluent

The diluent significantly influenced the stability of the chromogenic complex. Among the tested solvents including distilled water, ethanol, methanol, acetone, and acetonitrile, distilled water consistently produced the highest absorbance and best stability. Organic solvents, in contrast, reduced or disrupted complex formation. Consequently, distilled water was adopted as the diluent of choice for routine analysis.

### Stoichiometric analysis of the complex

**Fig. 6:**
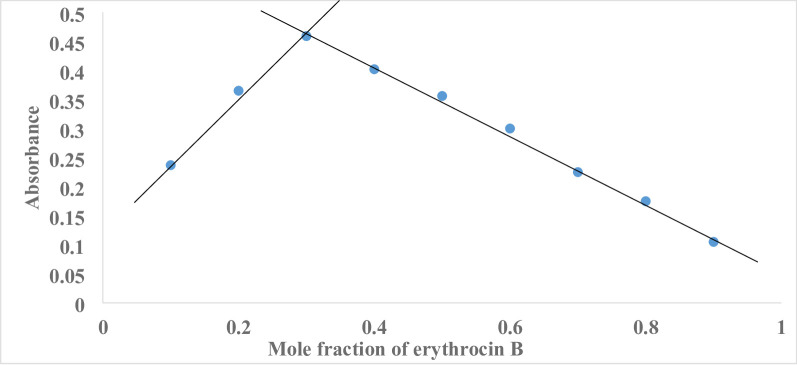
Job’s method using equimolar concentrations of both EB and MOX

The binding stoichiometry between MOX and EB was elucidated using Job’s method of continuous variation. Equimolar solutions of drug and dye were mixed in varying proportions while maintaining constant total volume. The absorbance profile revealed a maximum at a drug-to-dye ratio of 2:1, suggesting the formation of a stable complex with this composition (Fig. 6).

The observed 2:1 stoichiometric relationship indicates that two MOX molecules interact with a single EB molecule. This arrangement is consistent with the structural features of MOX as it contains two protonatable nitrogen atoms (one tertiary and one secondary) at position 7 of the quinolone nucleus. Under acidic conditions, both sites can be protonated, providing two cationic centers per MOX molecule. The negatively charged sulfonate groups of erythrosine B can therefore serve as a bridging locus, simultaneously interacting with two protonated MOX molecules.

Electrostatic attraction between the protonated nitrogen atoms of MOX and the sulfonate groups of EB appears to be the most plausible stabilizing force. Secondary hydrophobic interactions between the quinolone nucleus and the xanthene ring system of EB may also contribute to complex stability. These interpretations are based on spectrophotometric observations and structural features of MOX and EB but should be regarded as hypothetical rather than confirmed binding mechanisms. Future structural investigations using complementary techniques (FTIR, NMR, mass spectrometry, or computational modeling) are required to substantiate the proposed binding model.

### Method validation

Validation of the proposed spectrophotometric assay was carried out in accordance with ICH guidelines [31], ensuring its reliability for routine pharmaceutical analysis. The evaluation covered linearity, sensitivity, accuracy, precision, and robustness, each supported by experimental results.

All validation studies were performed in triplicate (*n* = 3) to ensure reproducibility. The limit of detection (LOD) and limit of quantification (LOQ) were calculated using the ICH formulas: LOD = 3.3σ/S and LOQ = 10σ/S, where σ is the standard deviation of the intercept and S is the slope of the calibration curve.

#### Linearity, detection, and quantification limits

The calibration curve for moxifloxacin over the range of 0.2–1.2 µg mL^− 1^ exhibited excellent linearity, with a correlation coefficient (*r*) of 0.9990 and determination coefficient (*r²*) of 0.9979 (Table 2). The regression equation showed a slope of 0.74 ± 0.02 and an intercept of 0.11 ± 0.01, confirming strong proportionality between absorbance and concentration. Sensitivity was excellent, with a limit of detection (LOD) of 0.05 µg mL^− 1^ and a limit of quantification (LOQ) of 0.15 µg mL^− 1^, highlighting the method’s ability to detect trace levels of MOX.

**Table 2:** Spectral features and validation parameters for the proposed spectrophotometric method

Parameters	Values
λ _max_ (nm)	554
Linear range (µg mL^− 1^)	0.2–1.2
Correlation coefficient (*r*)	0.9990
Determination coefficient (*r*^*2*^)	0.9979
Intercept ± SD*	0.11 ± 0.01
Slope ± SD	0.74 ± 0.02
Limit of Detection (µg mL^− 1^)^#^	0.05
Limit of Quantitation (µg mL^− 1^) ^#^	0.15
Molar absorptivity (ε, L·mol⁻¹·cm⁻¹)	3.41 × 10⁵
Sandell’s sensitivity (µg·cm⁻² per 0.001 AU)	1.18 × 10⁻³

SD*: Standard Deviation

^**#**^ LOD and LOQ values were calculated according to ICH guidelines using the formulas LOD = 3.3σ/S and LOQ = 10σ/S (*n* = 3)

In addition to ICH validation parameters, the molar absorptivity (ε) and Sandell’s sensitivity were calculated to further characterize the analytical performance of the proposed method. The molar absorptivity was found to be 3.41 × 10^5^ L·mol⁻¹·cm⁻¹, while Sandell’s sensitivity was 1.18 × 10^− 3^ µg·cm⁻² per 0.001 absorbance unit. These values confirm the high analytical sensitivity of the proposed method. Compared with previously reported spectrophotometric methods for moxifloxacin determination [22–26], the proposed MOX–EB assay demonstrates markedly superior analytical sensitivity. Earlier UV–Vis methods reported molar absorptivity values in the range of 0.5–1.2 × 10⁵ L·mol⁻¹·cm⁻¹ and Sandell’s sensitivity values around 3–6 × 10⁻³ µg·cm⁻² per 0.001 absorbance unit. In contrast, our method achieved a molar absorptivity of 3.41 × 10⁵ L·mol⁻¹·cm⁻¹ and Sandell’s sensitivity of 1.18 × 10⁻³ µg·cm⁻², underscoring its enhanced capability to detect trace levels of MOX. These findings highlight the improved sensitivity and eco-friendly design of the proposed assay, positioning it as a robust alternative to conventional spectrophotometric approaches.

#### Accuracy

Recovery studies at three concentration levels (0.4, 0.8, and 1.2 µg mL^− 1^) demonstrated mean recoveries of 99.77%, 99.95%, and 99.43%, respectively (Table 3). The relative standard deviation (RSD) values were consistently low (≤ 1.24%), confirming that the method provides true values without systematic bias.

**Table 3:** Evaluation of the accuracy of the proposed spectrophotometric method

Parameters	Mox Concentrations (µg mL^− 1^)		
	0.4	0.8	1.2
1	98.68	99	99.69
2	99.6	99.5	99.24
3	101.03	101.36	99.35
Mean	99.77	99.95	99.43
SD^*^	1.18	1.24	0.23
RSD (%) ^#^	1.18	1.24	0.23

^*^SD: Standard Deviation

^#^RSD: Relative Standard Deviation

#### Precision

Precision was evaluated through intra-day and inter-day studies. Intra-day recoveries ranged from 99.20% to 100.58%, while inter-day recoveries ranged from 99.35% to 100.52% (Table 4). The associated RSD values were below 1.72%, demonstrating excellent reproducibility across different days and experimental runs.

**Table 4:** Precision of the proposed spectrophotometric method

Concentration (µg mL^− 1^)	%Recovery *± SD	
	Intra-day	Inter-day
0.4	100.58 ± 1.39	99.35 ± 1.69
0.8	100.40 ± 1.35	100.52 ± 1.73
1.2	99.20 ± 0.46	99.83 ± 1.56

*Mean of three determinations

#### Robustness

Robustness was assessed by deliberately varying critical parameters such as buffer pH (3.3–3.5), buffer volume (1.8–2.2 mL), and dye volume (0.4–0.6 mL). Recovery values remained within 99.05–100.83% with low SD values (≤ 1.30) (Table 5), indicating that minor fluctuations in experimental conditions do not compromise the accuracy of the assay.

**Table 5:** Robustness for determination of MOX by the developed approach

Method parameters	%Recovery* ± SD
_*P*_H of acetate buffer	
3.3	99.05 ± 1.30
3.5	100.19 ± 0.36
Volume of acetate buffer (mL)	
1.8	99.95 ± 0.54
2.2	100.83 ± 0.58
EB volume	
0.4	99.14 ± 1.20
0.6	99.51 ± 0.84

*Mean of three determinations

### Application to pharmaceutical dosage forms

The method was successfully applied to Moxiflox^®^ eye drops, yielding recoveries between 99.10% and 100.46% across tested concentrations (0.4–1.2 µg mL^− 1^) (Table 6), with no evidence of signal distortion or anomalous absorbance. These findings confirm that the excipients present in the tested formulation did not interfere with the ion-pair complexation between MOX and EB.

**Table 6:** Recovery study for determination of MOX in Its eye drops formulation by the proposed spectrophotometric method

Dosage form	Conc taken (µg mL^− 1^)	Conc found (µg mL^− 1^)	Recovery (%) *± SD
Moxiflox^®^ drops	0.4	0.401	100.26 ± 0.88
	0.6	0.601	100.23 ± 1.87
	0.8	0.803	100.46 ± 0.41
	1.0	0.99	99.10 ± 0.27
	1.2	1.19	99.56 ± 0.29

*Mean of three determinations

The absence of interference was further supported by statistical comparison with the reported method [12] showed no significant differences, with calculated *t*-value (1.008) and *F*-value (1.124) both below the tabulated limits (2.306 and 6.338, respectively) at the 95% confidence level (Table 7). Overall, these results demonstrate the selectivity and robustness of the assay for routine analysis of ophthalmic dosage forms.

**Table 7:** Comparison of the results for analysis of Moxiflox^®^drops, the commercial dosage form of MOX, by proposed and reference method

Dosage form	% Recovery* ± SD		t-value^#^	F-value^#^
	Proposed method	Reported method		
Moxiflox^®^ Drops	100.49 ± 0.919	99.92 ± 0.86	1.008	1.124

*Mean is the average of five determinations

^#^Tabulated values at 95% confidence limit are *t*-value = 2.306, *F*-value = 6.338

### Greenness assessment of the proposed method

Evaluation of the environmental impact was conducted using three complementary tools: Analytical Eco-Scale, Green Analytical Procedure Index (GAPI), and Analytical GREEnness (AGREE) metrics.

**Fig. 7:**
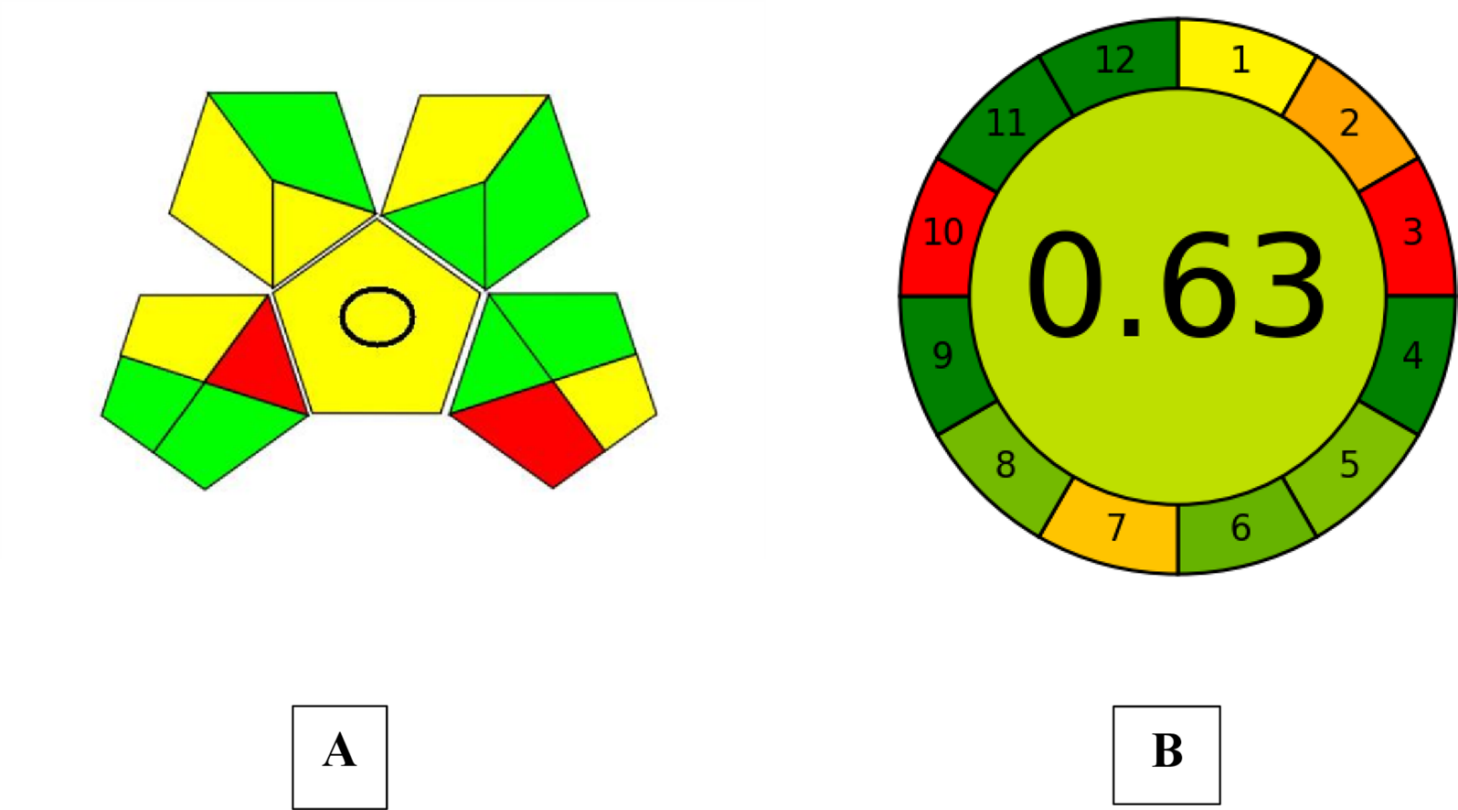
Evaluation of method greenness by (**A**) Green Analytical Procedure Index (GAPI), and **B** Analytical GREEnness (AGREE) metrics

- Eco-Scale: The total penalty points calculated for reagents, solvents, energy, waste, and occupational hazards amounted to be 13, yielding an Eco-Scale score of 87 (Table 8). This places the method in the category of excellent green analysis, reflecting the use of water as the sole solvent, minimal reagent consumption, and negligible energy requirements.
- GAPI: The GAPI pictogram (Fig. 7A) demonstrated predominantly green fields across sample preparation, reagents, instrumentation, and determination steps. Seven green field was observed, confirming compliance with green chemistry principles.

**Table 8:** Evaluation of proposed method greenness by eco-scale method

Item	Parameter	Word sign	PP score
Reagent(s)	Erythrosine B	LSH	1.0
	Sodium acetate buffer	MSH	6.0
Solvent	Water for dilution	LSH	0.0
Energy (kWh per sample)	˂ 0.1		0.0
Waste	1.0 − 10.0 mL		3.0 3.0
	No treatment		
Occupational hazards			0.0
(TPPs)			13.0
Eco-scale total score	= 100 – TPP		87.0

- AGREE: The AGREE evaluation produced an acceptable overall score (Fig. 7B), visually represented by a predominantly green circular diagram. This outcome highlights the method’s alignment with the principles of green analytical chemistry.

In contrast to conventional spectrophotometric methods that frequently employ hazardous solvents such as sulphuric acid, nitric acid, or hydrochloric acid, the proposed MOX–EB assay relies exclusively on water as the reaction medium. Conventional solvent-based procedures not only increase chemical consumption but also generate hazardous waste and pose occupational risks. By eliminating organic solvents, minimizing reagent volumes, and simplifying experimental steps, the present method achieves superior Eco-Scale, GAPI, and AGREE ratings, thereby demonstrating clear environmental advantages over traditional spectrophotometric approaches.

## Conclusion

The proposed spectrophotometric method for moxifloxacin determination via ion-pair complexation with EB has proven to be a robust, sensitive, and sustainable analytical tool. Validation studies confirmed excellent linearity (*r* = 0.9990), low detection and quantification limits (LOD = 0.05 µg mL^-1^, LOQ = 0.15 µg mL^-1^), and high accuracy with mean recoveries approaching 100%. Precision was consistently demonstrated, with intra-day and inter-day RSD values below 1.7%, while robustness testing showed that minor variations in buffer pH, buffer volume, or dye concentration did not compromise analytical performance. Application to commercial Moxiflox^®^ eye drops yielded recovery values between 99.10% and 100.46%, with statistical comparison to a reference method revealing no significant differences (*t* = 1.008, *F* = 1.124). These results establish the method as equivalent in accuracy and precision to established techniques, while offering simplicity, cost-effectiveness, and environmental compatibility.

The high molar absorptivity (3.41 × 10⁵ L·mol⁻¹·cm⁻¹) and low Sandell’s sensitivity (1.18 × 10⁻³ µg·cm⁻²) further emphasize the eco-friendly design of the MOX–EB assay, enabling trace-level detection of moxifloxacin without reliance on hazardous solvents and reinforcing its suitability for routine pharmaceutical quality control. By eliminating organic solvents, minimizing reagent consumption, and relying on accessible instrumentation, the assay aligns with green chemistry principles and offers a practical solution for routine pharmaceutical quality control, particularly in resource-limited laboratories. In essence, this validated method not only ensures drug quality and patient safety but also contributes to the broader goal of sustainable analytical practices in pharmaceutical sciences.

## Data Availability

All data supporting the findings of this study are included within the article.
